# Well-being and well-becoming through the life-course in public health economics research and policy: A new infographic

**DOI:** 10.3389/fpubh.2022.1035260

**Published:** 2022-12-23

**Authors:** Rhiannon Tudor Edwards

**Affiliations:** Centre for Health Economics and Medicines Evaluation (CHEME), Bangor University, Bangor, Gwynedd, United Kingdom

**Keywords:** public health, prevention, health economics, well-being, well-becoming, new infographic, life-course stages, economic evaluation

## Abstract

**Background:**

The term “well-becoming” is not new, but is not routinely used in our everyday language or in research in public health economics. It has been applied in early years research. Well-becoming can be thought of as our multitude of life-journeys toward meaning and purposefulness.

**Objective:**

To develop a new infographic in the spirit of the Dahlgren and Whitehead rainbow infographic of social determinants of health. The purpose being to redefine well-being as a process of growth through life, articulated as well-becoming.

**Methods:**

A rapid review of life-course stage appropriate models of well-being was undertaken with stages of the life-course as defined as: preconception and birth; early years; adolescence; working, parenting and caring; older age, and death. Infographics in this area were identified and the information above was used to design a new infographic with the concept of well-being and well-becoming at its center.

**Results:**

A new infographic reflecting an underlying concept of “the wheel of life” is presented. It shows movement through the life-course at its center, with concentric rings summarizing personal, local, and national and global factors that have an impact on well-being and well-becoming of individuals through the life-course. Of note, is the inclusion of death, which is a topic often avoided. Prepared during 2021–22, the infographic reflects the role of pandemic and war within the national and global ring of influential factors. I reflect on three ways in which health economists are currently using a life-course approach and the concept of well-becoming in the economic evaluation of individual programs and at a population level of government policy.

**Conclusion:**

Moving from solely focusing on a concept of well-being to a concept of well-being and well-becoming acknowledges the influence that socioeconomic and other conditions in a particular life-course stage have on subsequent life-course stages, and the cost-effectiveness of intervening across the life-course.

## Introduction

“Well-being” in the concept of population health can be thought of as “the balance point between an individual's resource pool and the challenges faced” (1). The World Health Organization (WHO) has promoted the concept of well-being since 1948 (2). There is still little consensus as to how it should be measured across many academic disciplines, including health economics, and the term is often used interchangeably with terms like “happiness” and “health-related quality of life” (3). Notably, however, the HM Treasury Green Book, update in 2021, has introduced the use of well-being adjusted life years (WELLBYs) based on life satisfaction measurement (4). In psychology and behavioral economics research, there is a distinction made between hedonic well-being and eudaimonic well-being (5). The hedonic viewpoint is based on the notion that increased pleasure and avoidance of pain leads to happiness (6). The eudaimonic viewpoint is defined more broadly to incorporate dynamic processes such as self-actualisation and the degree to which a person is fully functioning in society. Well-being is often made up of multiple components and is normative or subjective.

“Well-becoming” can be thought of as “our multitude of life-journeys toward meaning and purposefulness, not some steady-state of managed contentment” (7). The term well-becoming is not new, but is not a term we routinely use in our everyday language or in research in public health and health economics. Where it has appeared, it has predominantly been in early years research (8, 9). In an education context it has been used to carefully distinguish between how we help children in “being well” in the present and in well-becoming in shaping their future “being” and hence opportunities. In the field of health economics, the only place we have found reference to it is in the development of capability measures through the life-course (10). In mainstream economics, the financial benefits of investing in well-becoming are found in the work of James Heckman, a Chicago Nobel Prize-winning economist (11). Heckman demonstrated the life-time benefits of investing in high-quality pre-school care and the mitigating influence this can have on those with the worst start in life. Heckman has revised downwards some of his original estimates, but his arguments are still very powerful. Well-becoming is based on the concept of assets-based public health, which is concerned with identifying protective factors that support health and well-being (12).

Most references to the term “well-becoming” appear in research surrounding child development, for example, “in contrast to the immediacy of well-being, well-becoming describes a future focus (i.e., preparing children to be productive and happy adults, and to avoid social exclusion)” (13). Biggeri and Santi (14) discuss the concept of well-becoming in terms of system change in education to promote flourishing through a capability model aimed to support creativity, critical thinking and care.

It has been argued that concern for well-becoming should also be a concern for future generations (15). This links the concern for well-being with the concern for sustainable development and sustainable living (15). For policy makers and public health economists here in the UK, this attunes closely to the Well-being of Future Generations Act passed in Wales (16). In a later paper, Falkenberg (17) introduces the WB2-Framework for well-being and well-becoming. Falkenberg posits five components of well-being and well-becoming in an educational setting. These five components are: (1) Having Agentic Capabilities Linked to Human Needs; (2) Experiencing Situational Opportunities to Engage One's Agentic Capabilities; (3) Enjoying Life; (4) Living a Meaningful Life, and (5) Experiencing Personal and Communal Connections that Contribute to One's Well-being and Well-Becoming. Agentic refers to an individual's power to control his or her own goals, actions and destiny. In the US in 2021, the Youth Transition Funders Group made recommendations for investing in the well-being and well-becoming of America's young people (defined as the onset of puberty to the mid-20s) (18). They refer to well-being and well-becoming of young people in an equitable, inclusive, and holistic (whole person, whole life) context and offer a series of recommendations to transform public systems and communities to nurture and enhance lifelong well-being for vulnerable young people as they make the transition into adulthood. Again, from a UK public health economics perspective, this echoes emerging work on adverse childhood experiences and their life-time costs (19). The ethos of health economics is predicated on a societal goal of health gain, which implies health gain or reducing health losses from where we are now. In the same way, the concept of well-becoming is predicated on a goal of improving well-being (or minimizing potential well-being losses) in future life-course stages.

This paper presents a new infographic explaining the concept of well-becoming for the purpose of health economics research and policy support, within a life-course model. This new infographic builds on the much-loved Dahlgren-Whitehead (20) rainbow model of determinants of health. This paper moves on to present three ways in which health economists are currently taking a life-course approach to economic evaluation. First, the paper acknowledges that health economists have taken a life-time horizon in technical guidance produced by the National Institute for Health and Care Excellence (NICE) which calculates the absolute impact of an intervention across the life-course, but not necessarily the granular impact of different life-course stages (21). Second, the paper contrasts this with advances in the capability paradigm, based on Sen's contribution in development economics (22). The capability movement in health economics is producing life-course stage specific measures focusing on capabilities rather than quality-adjusted life years (QALYs) (10). Third, the paper illustrates calculation of costs of “late intervention” with case a study relating to dementia costs.

The paper sets out an agenda for research priorities in health economics to further the use of a well-becoming lens in the use of evidence in public policy. First, the use of differing time-horizons in economic evaluation of interventions to promote well-becoming needs to be routine. Second, there is a need for the acknowledgment of the need for intersectoral transfers where certain sectors pay to initiate well-becoming and others reap the financial benefits across the life-course. Finally, there is a need to explore how “money” can be put to work within the economy as a lever to engage effective and cost-effective interventions to promote well-being and well-becoming.

## Methods

**Figure 1:**
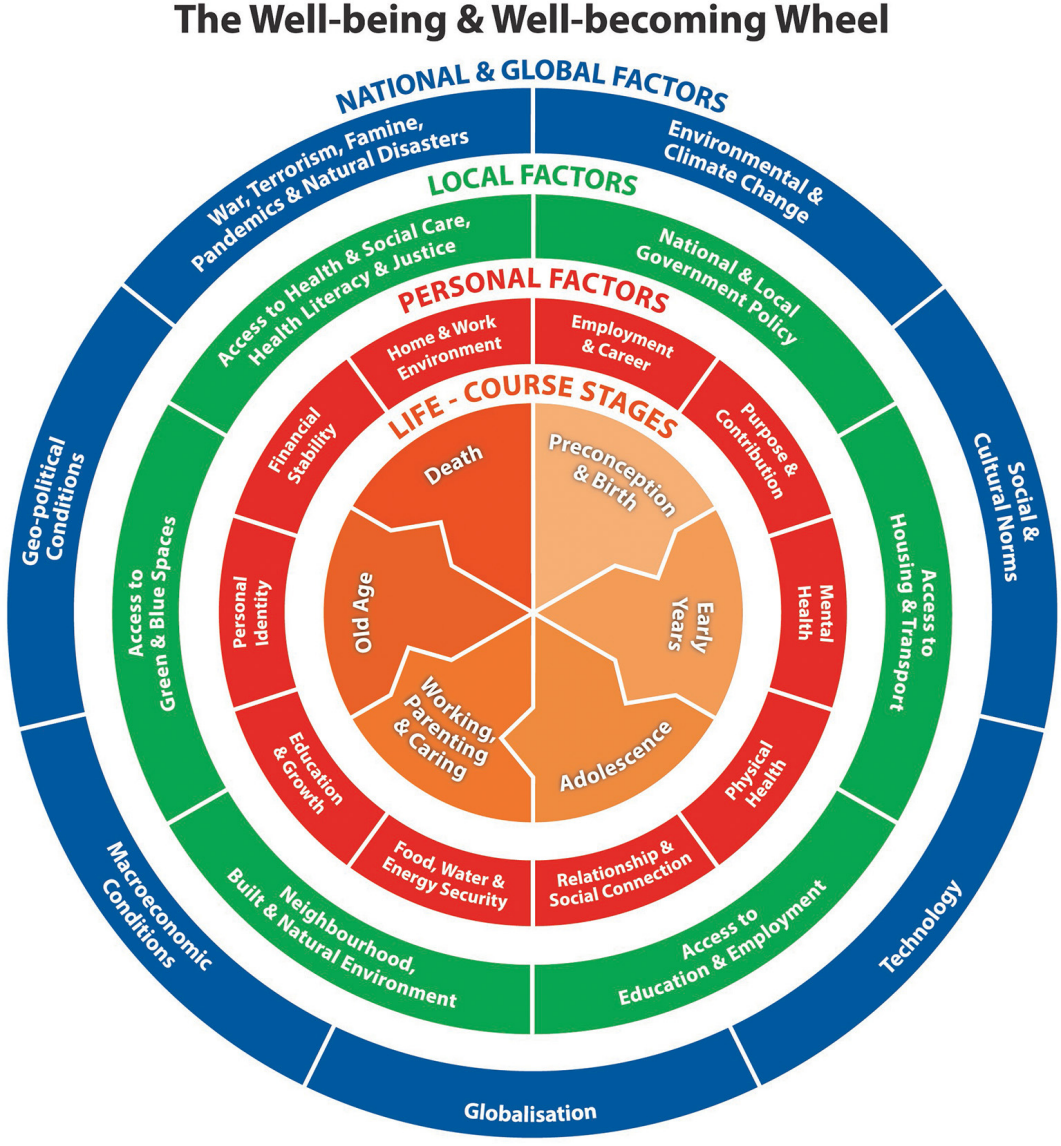
The well-being and well-becoming wheel.

Scoping reviews are used to identify knowledge gaps, set research agendas, and identify implications for decision-making (23). Using a scoping review methodology spanning the last 20 years), I reviewed models of the basic needs we all share across the life-course and how they constitute the wider determinants of our health, well-being and well-becoming. Based on this review, a new infographic was developed, as shown in Figure 1.

The new well-being and well-becoming wheel infographic was piloted with health economists working at the Center for Health Economics and Medicines Evaluation (CHEME) at Bangor University during 2022. The infographic is being translated into Welsh as it has been developed in a bilingual country.

## Results

### Development of a new infographic

In synthesizing the findings of the scoping review, I started with two overarching conceptual models and associated infographics. These were Maslow's (24) hierarchy of needs and the Dahlgren-Whitehead (20) rainbow model of health determinants. These appear as very static representations of human need and the wider determinants of health. They do not show any movement through the life-course. There seemed a need for a new infographic given the direction of many government policies increasingly taking a life-course approach to promoting well-being at local and national level. In 2018, The Health Foundation put forward a “road map” of what makes us healthy, referring to factors such as good work; our surroundings; money and resources; housing; education and skills; the food we eat; transport, and family, friends and communities (25). This model helped shape the red inner ring of the new infographic shown in Figure 1.

A number of models of the factors that influence well-being have been put forward, which are life-course stage specific. For example, UNICEF (26) identified six dimensions of child well-being, which are: material well-being; health and safety; educational well-being; family and peer relationships; behaviors and risks, and subjective well-being. An example of a model of well-being in adolescence was put forward by Ross et al. (27). This model is made up of five domains, which are: good health and optimum nutrition; connectedness, positive values, and contribution to society; safety and a supportive environment; learning, competence, education, skills, and employability, and agency and resilience. A model of factors determining well-being in the workplace was put forward by the Chartered Institute of Personnel and Development (28). These factors span: pay and benefits; contracts; work-life balance; job design and the nature of work; relationships at work; employee voice, and health and well-being. With respect to promoting well-being in older age, the World Health Organization (29) put forward a model of factors necessary for age-friendly living. These span: housing; social participation; respect and social inclusion; civic participation and employment; communication and information; community support and health services; outdoor spaces and buildings, and transportation. Many of these factors are represented in the second concentric ring on the new infographic, labeled as local factors. A recent model of factors that can influence what might be considered a good death is offered by Campbell (30). This includes: place of death; one's company in death; cause of death, and one's manner of facing death.

Figure 1 shows the new infographic entitled “The well-being and well-becoming wheel”. This infographic reflects an underlying concept of “the wheel of life”. The wheel of life is a cyclic representation of life found on the walls of Tibetan Buddhist temples and monasteries (31). The infographic shows movement through the life-course at its center, with concentric rings summarizing personal, local, and national and global factors that have an impact on well-being and well-becoming on individuals through the life-course. The choice of the life stages has come from a review of existing models in the literature (25–30).

Of note, is the inclusion of death, which is a topic often avoided in the discussion of life-course stages (32). Prepared during 2021–22, the infographic reflects the role of pandemic and war within the international and global ring of influential factors. The first concentric ring is red and reflects personal factors that determine or have an impact through the life-course on well-being and well-becoming. These are: employment and career; purpose and contribution; mental health; physical health; relationship and social connection; recreation and fun; education and growth; personal identity; financial stability, and home and work environment. Of note is the inclusion of “Purpose and Contribution” to reflect the work of Paul Dolan who describes happiness as experiences of pleasure and purpose over time (33). The second concentric ring is green and reflects local factors that determine or have an impact on well-being and well-becoming through the life-course. These are: national and local government policy; access to housing and transport; access to education and employment; neighborhood, built and natural environment; access to green and blue spaces; access to health and social care, health literacy and justice. Neighborhoods are explicitly mentioned to reflect the emphasis placed on neighborouhood environments as key determinants of the health and life expectancy gradient by Michael Marmot in “The Marmot Review” and “The Marmot Review 10 Years On” reports (34, 35). The third concentric ring is blue and reflects national and global factors that determine or have an impact on well-being and well-becoming through the life-course. These are: environmental change (e.g., climate change); social and cultural norms; technology; globalization; macroeconomic conditions; geo-political conditions, and war, terrorism, famine, pandemics and natural disasters.

### Example of a life-course stage approach to public health prevention—dementia

Livingston et al. (36) in a Lancet Commission report set out 12 potentially modifiable risk factors for dementia. I use this as an example for taking a life-course approach to public health policy for a specific condition. Table 1 shows what percentage of prevalence of dementia could be reduced by eliminating 12 risk factors across the life-course. These are: limited or less education; hearing loss; traumatic brain injury; hypertension; alcohol (>21 units per week); obesity; smoking; depression; social isolation; physical inactivity; air pollution, and diabetes. This report used three life-course stages and relates the above risk factors to life, midlife, and later life. Overall, Livingston et al. conclude that 40% of risk factors may be modifiable earlier in the life-course, while 60% are at present unknown.

**Table 1:** Modifiable risk factors for dementia by life stage [adapted from Livingston et al. (36)].

**Life stage**	**Risk factor**	**Percentage reduction in dementia prevalence if risk factor is eliminated (%)**
Early life	Less education	7
Midlife	Hearing loss	8
	Traumatic brain injury	3
	Hypertension	2
	Alcohol >21 units per week	1
	Obesity	1
Later life	Smoking	5
	Depression	4
	Social isolation	4
	Physical inactivity	2
	Air pollution	2
	Diabetes	1
Sum of potentially modifiable risk factors		40
Unknown risk factors		60

### Current use of a life-course approach to well-being and well-becoming in health economics

Health economics has evolved over the last 60 years within a medical, evidence-based model of health. Within this medical model of health, NICE has promoted use of a lifetime horizon in health technology assessment in its reference case for the evaluation of cost-effectiveness (21). Most economic evaluation studies are of single interventions which form part of pathways of care for patients and are treatment or cure focused. The application of health economics to public health requires a far more broad perspective of analysis with which to view costs and outcomes, and a far more diverse toolbox of methods of analysis spanning cost benefit analysis and social return on investment analysis through to econometric modeling at a population level. Hence, just as there has been a move to a more socioeconomic conceptualization of health, I am amongst other health economists who have aligned themselves with such a paradigm shift in health economics. This has been in terms of public health economics (37) and in terms of addressing equity considerations in cost-effectiveness analysis (38).

As a challenge to the QALY used by NICE, there is a programme of work developing in the capability paradigm of outcome measurement in health economics. This centers around a framework based on conceiving capabilities as evolving across the life-course (10). Concerning children and young people, the term well-being capability is used. There is definitely a synergy of ideas evolving and the need for expanding the evaluative space beyond health functioning toward broader capabilities is very relevant to my understanding of well-being and well-becoming. The capability movement in outcome measurement in health economics is addressing the challenge of measuring outcomes within a life-course model. This approach allows for the development of an economic evaluation framework that places different capabilities at the center of attention depending on where an individual is at in their life-course trajectory (10). A recent paper by Deidda et al. (39) is a good example of a cost-effectiveness analysis of a public health prevention interventionwhich used both QALYs and well-being capabilities in the area of reducing sedentary behavior across four European countries. It is still useful to have evidence of the cost per QALY of preventative interventions as this allows for comparison with the cost per QALY of new medicines routinely funded by the NHS. It has been estimated that 80 percent of chronic health problems, such as premature heart disease, stroke and diabetes, are preventable (40).

A life-course approach to dynamic microsimulation modeling can support long-term thinking in the development and evaluation of government policy particularly with respect to the early years. Skarda et al. (41) present such an example of a dynamic microsimulation model for childhood policy analysis that models developmental, economic, social and health outcomes from birth to death for each child in the Millennium Cohort Study (MCS) in England, together with public costs and a summary well-being measure. The model draws on observational data from the MCS focusing on health, conduct disorder, mortality, health-related quality of life, public costs and a general well-being metric. The paper includes a discussion of the shortcomings of unweighted benefit cost analysis and alternatives including utilitarian and priortarian approaches to economic evaluation based on explicitly individual well-being and social welfare functions (38).

It may be that it is not single cost-effectiveness studies that have the biggest impact on shifting emphasis from “cure to prevention”, but rather the availability of a growing body of evidence on cost-effectiveness and return on investment that effectively shifts thinking in political circles toward a longer time horizon for policy. This was perhaps encapsulated as far back as 1977 when Weiss (42), an American scholar of education and policy analysis, wrote: “the major use of social research is not the application of specific data to specific decisions. Rather, government decision makers tend to use research indirectly, as a source of ideas, information, and orientations to the world. Although the process is not easily discernible, over time it may have profound effects on policy. Even research that challenges current values and political feasibilities is judged useful by decision makers.”

Another approach in health economics which could be characterized by a life-course approach is the calculation of costs of the “late intervention” in childhood problems. Chowdry and Fitzsimmons (43) estimated the financial sum spent at local authority level across England and Wales as a result of late intervention in early childhood problems. Of the total annual spend of £16.6 billion in 2016–17 prices, £6.4 billion was spent by local government (39%), £3.7 billion was spent by the NHS (22%); £2.7 billion was spent by the Department for Work and Pensions (16%); £1.6 billion was spent by the police (10%), £1.5 billion was spent by the criminal justice system (9%), and £655 million was spent by education (4%).

## Discussion

Michael Marmot has argued that: “Given the attack on science by politicians of bad faith, it is important to recognize that epidemiology and public health have a crucial role to play in providing evidence to improve health of society and reduce inequalities” (44). This paper began by presenting a working definition of well-being and well-becoming. The concept of well-becoming is not new, but it is not a word we use very frequently in discussions about public health and health economics. It embodies the concept of “flow” and “growth” through the life-course. Obviously, trajectories through the life-course are not always positive and can fluctuate. However, these concepts support the design and development of public health policy and the way we go about economic evaluation of such policies by providing a dynamic lens, rather than a static lens, and inherently changing our time horizon and perhaps our time preference in tackling health and social problems.

I have not found any examples of publications that, prior to this paper, examine side-by-side models of the determinants of well-being at different life-course stages. This was done as a precursor to the design of the new well-being and well-becoming infographic, presented here in Figure 1. At the time of writing, world leaders were meeting at COP27 and I came across, late in the day, an infographic known as Doughnut Economics, a model of human well-being which recognizes that well-being depends on enabling every person to lead a life of dignity and opportunity, while safeguarding the integrity of Earth's life-supporting systems (45). In some senses, the infographic developed here for the field of health economics complements the work of Raworth (45).

This new Well-being and Well-becoming Wheel infographic does not attempt to convey directions for causality or feedback loops whilst it recognizes that these are complex and many. Neither does it attempt to convey the fact that the central life-course stages can sometimes be overlapping and are very different from individual to individual. Instead, it attempts to convey the concept of flow, growth and well-becoming through life as a basis for how we approach the design of public health interventions, evaluate their effectiveness and cost-effectiveness, and set overarching priorities for public spending (37).

In the evaluation of specific diagnostic and therapeutic interventions, using the NICE (21) reference case, health economists are using a life-course approach in terms of time horizon, but arguably not in terms of a wider life-course trajectory context. In addition to this, some health economists are developing capabilities-based outcome measures as a challenge to the QALY paradigm. Health economists taking a life-course approach in population level econometric modeling of government policy. And health economists are beginning to quantify the cost of late intervention.

This infographic has been prepared as part of a new book exploring the health economics of well-being and well-becoming through the life-course to be published by Oxford University Press in 2023. The book argues that with an agenda for research priorities in health economics to further the use of a well-becoming lens in the use of evidence in public policy. These priorities include use of differing time-horizons in economic evaluation of interventions to promote well-becoming; acknowledgment of the need for intersectoral transfers where certain sectors pay to initiate well-becoming and others reap the financial benefits across the life-course, and finally, how “money” can be put to work within the economy as a lever to engage effective and cost-effective interventions to promote well-being and well-becoming in society.

## Conclusion

Health economists in the field of public health have a crucial role given the relatively short time-horizon of politics and local planning to provide evidence of both short term and longer term costs and outcomes of using resources in different ways to address population health priorities (37). A life-course lens on well-being and well-becoming applied to health economics research routinely may in time help to shift the focus of policy further toward commitment to a prevention agenda. This is the first time that this new well-being and well-becoming infographic has been published and it is hoped that it can be a useful addition to emerging debate.

## Author contributions

RTE conceived this paper, undertook the research, and the infographic was her idea.
